# Rising Population Cost for Treating People Living with HIV in the UK, 1997-2013

**DOI:** 10.1371/journal.pone.0015677

**Published:** 2010-12-30

**Authors:** Sundhiya Mandalia, Roshni Mandalia, Gary Lo, Tim Chadborn, Peter Sharott, Mike Youle, Jane Anderson, Guy Baily, Ray Brettle, Martin Fisher, Mark Gompels, George Kinghorn, Margaret Johnson, Brendan McCarron, Anton Pozniak, Alan Tang, John Walsh, David White, Ian Williams, Brian Gazzard, Eduard J. Beck, for the NPMS-HHC Steering Group

**Affiliations:** 1 NPMS-HHC Coordinating and Analytic Centre, London, United Kingdom; 2 Imperial College, London, United Kingdom; 3 Chelsea and Westminster Hospital, London, United Kingdom; 4 Health Protection Agency, London, United Kingdom; 5 London Specialised Commissioning Group, London Procurement Programme, London, United Kingdom; 6 Royal Free Hospital, London, United Kingdom; 7 Homerton University Hospital NHS Foundation Trust, London, United Kingdom; 8 London and Barts Hospitals, London, United Kingdom; 9 Edinburgh General Hospital, Edinburgh, United Kingdom; 10 Royal County Sussex Hospital, Brighton, United Kingdom; 11 Southmead Hospital, Bristol, United Kingdom; 12 Royal Hallamshire Hospital, Sheffield, United Kingdom; 13 James Cook University Hospital, Middlesborough, United Kingdom; 14 Royal Berkshire Hospital, Berkshire, United Kingdom; 15 St. Mary's Hospital, London, United Kingdom; 16 Birmingham Heartlands Hospital, Birmingham, United Kingdom; 17 Mortimer Market Centre, London, United Kingdom; 18 London School of Hygiene & Tropical Medicine, London, United Kingdom; University of Toronto, Canada

## Abstract

**Background:**

The number of people living with HIV (PLHIV) is increasing in the UK. This study estimated the annual population cost of providing HIV services in the UK, 1997–2006 and projected them 2007–2013.

**Methods:**

Annual cost of HIV treatment for PLHIV by stage of HIV infection and type of ART was calculated (UK pounds, 2006 prices). Population costs were derived by multiplying the number of PLHIV by their annual cost for 1997–2006 and projected 2007–2013.

**Results:**

Average annual treatment costs across all stages of HIV infection ranged from £17,034 in 1997 to £18,087 in 2006 for PLHIV on mono-therapy and from £27,649 in 1997 to £32,322 in 2006 for those on quadruple-or-more ART. The number of PLHIV using NHS services rose from 16,075 to 52,083 in 2006 and was projected to increase to 78,370 by 2013. Annual population cost rose from £104 million in 1997 to £483 million in 2006, with a projected annual cost between £721 and £758 million by 2013. When including community care costs, costs increased from £164 million in 1997, to £683 million in 2006 and between £1,019 and £1,065 million in 2013.

**Conclusions:**

Increased number of PLHIV using NHS services resulted in rising UK population costs. Population costs are expected to continue to increase, partly due to PLHIV's longer survival on ART and the relative lack of success of HIV preventing programs. Where possible, the cost of HIV treatment and care needs to be reduced without reducing the quality of services, and prevention programs need to become more effective. While high income countries are struggling to meet these increasing costs, middle- and lower-income countries with larger epidemics are likely to find it even more difficult to meet these increasing demands, given that they have fewer resources.

## Introduction

The UK has the fastest growing HIV epidemic in Western Europe [1]. The number of PLHIV alive and using NHS services have increased, partly due to more effective ART regimens resulting in their longer survival [2], partly due to uninfected people continuing to be infected with HIV either in the UK or abroad [3]. This combination has resulted in increasing number of PLHIV using NHS services [4] which is likely to have resulted in increased population costs for HIV treatment and care. The aim of this study was to estimate the treatment and care costs for PLHIV in the UK population by stage of HIV infection and type of ART between 1997 and 2006 and project costs for 2007–2013.

## Methods

The National Prospective Monitoring System on the use, cost and outcome of HIV service provision in UK hospitals - HIV Health-economics Collaboration (NPMS-HHC) monitors prospectively the effectiveness, efficiency, equity and acceptability of treatment and care in participating HIV units since 1996. Using an agreed minimum dataset, standardized data are routinely collected in clinics and transferred to the NPMS-HHC Coordinating and Analytic Centre, ensuring both patient and clinic confidentiality. The data analyses are performed both at clinic and aggregate levels. The clinic specific analyses remain confidential, while aggregate analyses become public documents [5], [6].

### Use and Cost of Hospital Services

Information on the use of hospital inpatient (IP), outpatient (OP), and dayward (DW) services between 1^st^ January 1997 and 31^st^ December 2006, was obtained from 14 hospitals participating in this analysis. The mean number of IP days, OP and DW visits per patient-year (PPY) by stage of HIV infection - asymptomatic, symptomatic non-AIDS or AIDS - were calculated per patient-year from 1997 to 2006. The methods used for calculating the mean use of hospital services per patient year (PPY) were similar to those employed in our previous studies [2], [7], [8].

The denominator consisted of the total duration of follow up for all patients during a calendar year, from when they were first seen during that year till the end of the year if still alive, or when they died, or if they were lost to follow up, which ever came first. Numerators were calculated by summing the use of IP, OP or DW services and annual mean use of services PPY were calculated using the following formula:
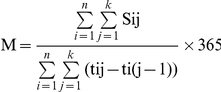



Where: n  =  total number of individuals;

k  =  day of censoring;

S_ij_  =  use of service of individual i at jth day;

t_ij_  =  number of days since diagnosis of stage of HIV infection of individual i and remaining within the same stage;

M  =  mean of services S per patient-year by stage of HIV infection.

Annual cost PPY estimates for HIV-service provision for individual PLHIV by stage of infection were produced by linking mean number of IP days, OP visits and DW visits with their respective unit costs (2006 prices). The unit cost estimates for an average IP day was £475, average OP visit was £94 and £384 for a DW visit [9]. Costs were in UK pounds at 2006 prices.

### Unit Costs of Antiretroviral Therapy

Average costs for treatment regimens were based on London Specialised Commissioning Group prices of each licensed antiretroviral (ARV) drug. Average annual cost, including 17.5% value added tax (VAT), were calculated for different antiretroviral therapy (ART) regimens - mono-, dual-, triple- or quadruple-or-more therapy - and stratified by stages of HIV infection: asymptomatic, symptomatic non-AIDS or AIDS.

### Overall Cost of Services by Stage of HIV Infection and Type of Antiretroviral Therapy

The costs for different ART by stage of HIV infection were added to the cost for IP, OP and DW services for each of the clinical stages; costs of ‘other’ drugs and tests and procedures performed [9], were added to obtain the total direct costs for treatment and care for PLHIV by stage of HIV infection and type of ART. The primary analysis was from a public service perspective focused on hospital costs [10], while annual population cost-estimates were also presented including community care costs [9] as indicated.

### PLHIV Using NHS Services by Type of Antiretroviral Therapy

The annual number of PLHIV by stage of HIV infection using NHS services by type of ART prescribed, were obtained from the Health Protection Agency (HPA). Not all of these data sets were complete; for some PLHIV their stage of HIV infection was known but the type of ART prescribed was unknown. These individuals were proportionally distributed across the respective ART strata, ensuring that the proportion of subjects represented in each category remained unchanged. Secondly, HPA figures indicated that no subjects had been prescribed quadruple-or-more therapy in 1997 and 1998, which was unlikely and probably due to incorrect reporting during those calendar years. For these years linear least squares regression analysis [11] was used to estimate the number of PLHIV likely to have been prescribed quadruple- or-more therapy.

### UK HIV Population Cost Estimate

To obtain the population cost estimates, the total annual treatment and care costs for a PLHIV by stage of HIV infection and type of ART were multiplied by the number of PLHIV within those categories for each year. Costs were then added across stages of HIV and types of ART regimens to obtain a total population cost by year, while community care costs were also included for population estimates [9].

### UK HIV Population and Cost Projection

Projections were made to estimate future costs for the years 2007 to 2013 and these were investigated using both the linear and higher order polynomial least squares regression analyses [11]. Using proportion of variance explained as a measure of goodness of fit of the models, the linear least squares analyses produced the best fit and was therefore presented in this paper. The projections were based on the trends observed over 1997–2006 and three scenarios were investigated: the *first* scenario extrapolated total annual population costs 1997–2006 to 2007–2013. The *second* scenario extrapolated the total number of PLHIV who used HIV services between 1997 and 2006 to 2007 and 2013. The average annual cost of treating a PLHIV across all stages of HIV infection in 2006 was calculated and this was multiplied by the projected numbers of PLHIV using NHS services for the years 2007–2013. The *third* scenario extrapolated PLHIV for each of the three stages of HIV infection from 1997–2006 to 2007–2013. Average 2006 annual cost of treatment and care for stage of HIV infection was used to extrapolate cost 2007–2013 by stage of HIV infection. Total projected annual treatment costs were obtained by adding annual population costs of each of the three stages of HIV infection across all three stages.

## Results

26,033 PLHIV were managed in 14 UK HIV centres between 1997 and 2006. Seventy six percent of the study sample were men and 51% of all PLHIV were Caucasian. Of those whose sexual orientation was known, 62% were men who have sex with men (MSM), while 4.5% were known to be or have been injecting drug users (IDUs). Seventy nine percent of the HIV subjects had attended HIV centres in London for HIV care, while 15% were known to have attended more than one HIV centre within the UK during the study period.

### Use of Hospital Services, 1997–2010

#### Annual Mean Number of Inpatient Days

**Figure 1:**
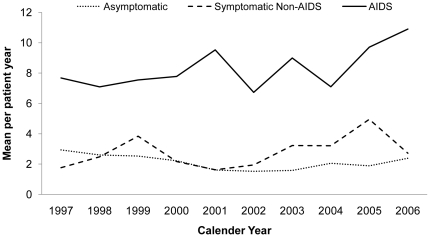
Mean Inpatient Days per Patient-year by Stage of HIV Infection and Year.

Average mean number of IP days during the study period was 2.1 PPY for asymptomatic patients, which did not change substantially over time. Average mean number of IP days for symptomatic non-AIDS patients increased from 1.8 in 1997 to 2.7 PPY in 2006, while the average number of IP days for AIDS patients decreased from 7.7 in 1997 to 6.7 PPY in 2002 but increased to 10.9 in 2006 (Figure 1).

#### Annual Mean Number of Outpatient Visits

**Figure 2:**
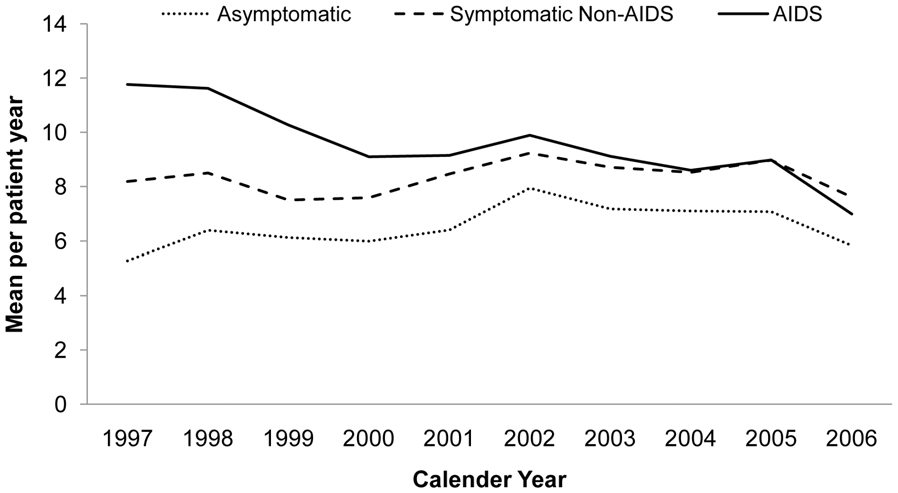
Mean Outpatient Visits Per Patient-year by Stage of HIV Infection and Year.

Average mean number of OP visits for asymptomatic patients was 6.5 PPY which did not change substantially over time, nor did the average mean number of OP visits for symptomatic non-AIDS patients at around 8.3 PPY. For AIDS patients, the mean number of OP visits decreased from 11.8 in 1997 to 7.0 PPY in 2006 (Figure 2).

#### Annual Mean Number of Dayward Visits

**Figure 3:**
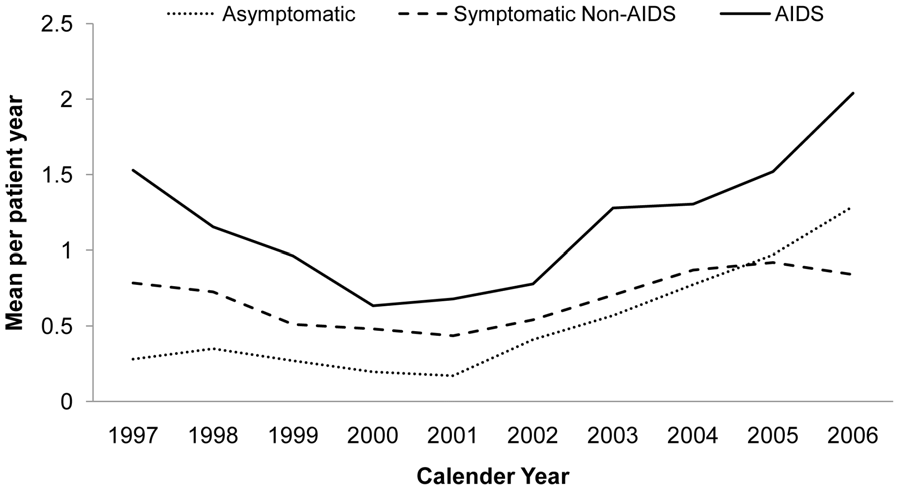
Mean Dayward Visits Per Patient-year by Stage of HIV Infection and Year.

The average mean number of DW visits for asymptomatic patients increased from 0.3 in 1997 to 1.3 PPY in 2006. The average mean number of DW visits for symptomatic non-AIDS patients remained constant at 0.7 PPY, while for AIDS patients the average mean DW visits increased from 1.5 in 1997 to 2.0 PPY in 2006, having first decreased to 0.6 in 2000 (Figure 3).

### Annual Cost of Treatment and Care Services by Type of ART

Treatment and care costs for PLHIV at different stage of HIV infection and different treatment regimens increased over time (Table 1).

**Table 1:** Annual cost of treatment and care by Stage of HIV Infection and different types of Anti-Retroviral Therapy in UK pound, 2006 UK prices and proportion of cost due to Antiretroviral Therapy (%).

Year	Monotherapy			Dual therapy			Triple therapy			Quadruple or more therapy		
	Asymp-tomatic	SymptomaticNon AIDS	AIDS	Asymp-tomatic	SymptomaticNon AIDS	AIDS	Asymp-tomatic	SymptomaticNon AIDS	AIDS	Asymp-tomatic	SymptomaticNon AIDS	AIDS
**1997**	£8,308	£12,076	£30,717	£11,376	£14,685	£33,726	£16,347	£19,806	£38,887	£18,839	£22,724	£41,383
	(46%)	(36%)	(13%)	(61%)	(47%)	(21%)	(73%)	(61%)	(31%)	(76%)	(66%)	(35%)
**1998**	£8,642	£13,008	£29,609	£11,660	£15,907	£32,467	£16,542	£21,006	£37,626	£19,528	£23,970	£40,621
	(48%)	(34%)	(15%)	(62%)	(46%)	(22%)	(73%)	(59%)	(33%)	(77%)	(64%)	(38%)
**1999**	£8,552	£13,481	£30,431	£11,619	£16,553	£33,542	£16,506	£21,980	£38,483	£19,894	£25,140	£42,005
	(50%)	(29%)	(14%)	(63%)	(43%)	(22%)	(74%)	(57%)	(32%)	(78%)	(62%)	(38%)
**2000**	£8,083	£11,299	£29,274	£10,981	£14,352	£32,423	£15,941	£19,683	£37,242	£18,734	£22,476	£40,380
	(51%)	(33%)	(14%)	(64%)	(47%)	(22%)	(75%)	(62%)	(32%)	(79%)	(66%)	(38%)
**2001**	£7,859	£11,489	£32,229	£10,906	£14,310	£35,493	£15,827	£19,521	£39,981	£20,613	£23,709	£44,911
	(52%)	(35%)	(13%)	(65%)	(47%)	(21%)	(76%)	(62%)	(30%)	(82%)	(68%)	(37%)
**2002**	£8,145	£11,901	£29,059	£11,358	£14,314	£32,164	£16,257	£19,954	£36,978	£21,449	£26,414	£42,235
	(49%)	(35%)	(14%)	(64%)	(46%)	(22%)	(75%)	(61%)	(32%)	(81%)	(71%)	(41%)
**2003**	£8,176	£13,435	£32,227	£11,319	£15,611	£35,454	£16,422	£21,474	£40,221	£21,747	£26,089	£45,831
	(49%)	(34%)	(13%)	(64%)	(43%)	(21%)	(75%)	(59%)	(30%)	(81%)	(66%)	(39%)
**2004**	£8,774	£12,272	£28,988	£11,777	£15,483	£32,206	£17,254	£21,621	£37,311	£22,827	£26,659	£43,436
	(48%)	(28%)	(15%)	(61%)	(43%)	(23%)	(74%)	(59%)	(34%)	(80%)	(67%)	(43%)
**2005**	£8,685	£12,192	£30,566	£11,864	£17,048	£33,864	£16,930	£22,713	£39,045	£22,979	£28,603	£45,661
	(50%)	(17%)	(14%)	(64%)	(41%)	(22%)	(74%)	(56%)	(32%)	(81%)	(65%)	(42%)
**2006**	£10,020	£11,482	£32,760	£13,351	£15,692	£36,580	£18,280	£21,597	£41,747	£23,775	£25,135	£48,055
	(43%)	(23%)	(11%)	(58%)	(43%)	(20%)	(69%)	(59%)	(30%)	(76%)	(72%)	(39%)

#### Mono-therapy

For asymptomatic individuals in 1997 the annual cost of care was £8,308, which increased to £10,020 in 2006. For those with symptomatic non-AIDS, the 1997 cost of £12,076 decreased to £11,487, while for AIDS patients on mono-therapy, the annual costs increased from £30,717 in 1997 to £32,760 by 2006.

#### Dual-therapy

For asymptomatic PLHIV on dual therapy, the 1997 cost of £11,376 increased to £13,351. For patients with symptomatic non-AIDS, the annual cost increased from £14,685 in 1997 to £15,692 in 2006, while for AIDS patients on dual therapy, this increased from £33,726 in 1997 to £36,580 per patient-years in 2006.

#### Triple-Therapy

For asymptomatic PLHIV on triple therapy, annual costs increased from £16,347 to £18,280 in 2006. For patients with symptomatic non-AIDS annual costs increased from £19,806 to £21,597 in 2006, while for AIDS patients annual costs varied from £38,887 in 1997 to £41,747 in 2006.

#### Quadruple-or-more-Therapy

Annual cost for those asymptomatic PLHIV on quadruple-or-more therapy ranged from £18,839 in 1997 to £23,775 in 2006. Similarly annual costs increased from £22,724 to £25,135 for PLHIV with symptomatic non-AIDS, while for AIDS patients annual costs increased from £41,383 to £48,055 in 2006.

It was of interest to note that the proportion spent on ART did not change significantly over time for any of three stages of HIV infection nor for the four ART treatment categories (Table 1).

### Population Cost Treatment and Care 1997–2006 and 2007–2013

#### People Living With HIV Using NHS Services

The total number of PLHIV who used NHS services for treatment and care increased from 16,075 patients in 1997 to 52,083 by 2006, an increase that was seen across all stages of HIV infections. However, the proportion of asymptomatic PLHIV increased from 38% to 49% between 1997 and 2006, with the proportion of symptomatic non-AIDS patients and AIDS patients both decreasing, from 34% to 28% and from 28% to 23% respectively between 1997 and 2006 (Table 2).

**Table 2:** Number of People Living with HIV Using NHS Services by Stage of HIV 1997–2006 [4] and Projected Figures for 2007–2013 (in italics).

Year	HIV Population (%)			
	Asymptomatic	Symptomatic non-AIDS	AIDS	Total
**1997**	6,124 (38%)	5,384 (34%)	4,567 (28%)	16,075
**1998**	5,835 (32%)	6,975 (38%)	5,525 (30%)	18,335
**1999**	6,833 (33%)	7,994 (37%)	6,288 (30%)	21,114
**2000**	8,055 (35%)	8,347 (37%)	6,338 (28%)	22,740
**2001**	10,015 (38%)	9,088 (34%)	7,253 (28%)	26,356
**2002**	12,787 (40%)	10,635 (34%)	8,115 (26%)	31,537
**2003**	15,932 (43%)	11,544 (32%)	9,203 (25%)	36,679
**2004**	18,794 (45%)	12,834 (31%)	10,009 (24%)	41,637
**2005**	21,656 (46%)	14,590 (31%)	10,779 (23%)	47,025
**2006**	25,385 (49%)	14,750 (28%)	11,947 (23%)	52,083
**2007**	*25,485 (47%)*	*15,979 (30%)*	*12,378 (23%)*	*53,842*
**2008**	*27,729 (48%)*	*17,027 (29%)*	*13,173 (23%)*	*57,930*
**2009**	*29,974 (48%)*	*18,075 (29%)*	*13,969 (23%)*	*62,018*
**2010**	*32,218 (49%)*	*19,123 (29%)*	*14,764 (22%)*	*66,106*
**2011**	*34,462 (49%)*	*20,171 (29%)*	*15,560 (22%)*	*70,194*
**2012**	*36,707 (49%)*	*21,219 (29%)*	*16,355 (22%)*	*74,282*
**2013**	*38,951 (50%)*	*22,267 (28%)*	*17,151 (22%)*	*78,370*

#### Annual Population Costs 1997–2006

**Figure 4:**
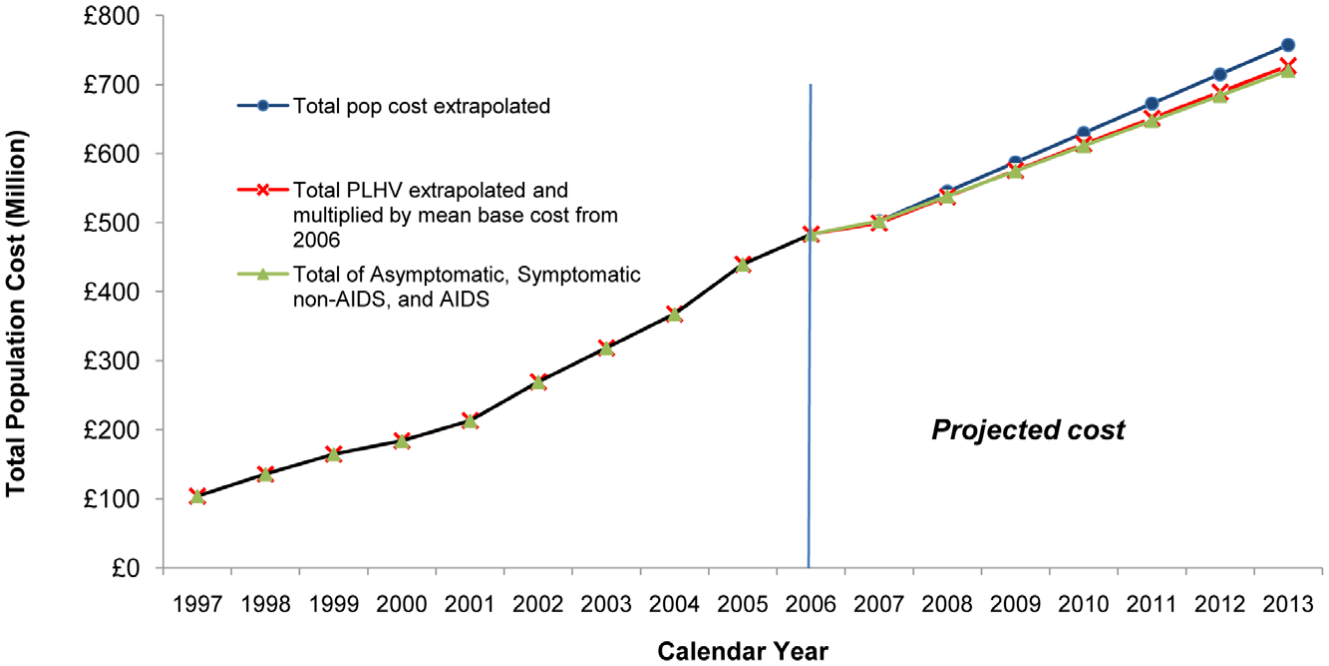
Annual Average UK Direct Population Cost 1997–2006 and Projections 2007–2013 based on three Scenarios (UK 2006 prices).

**Figure 5:**
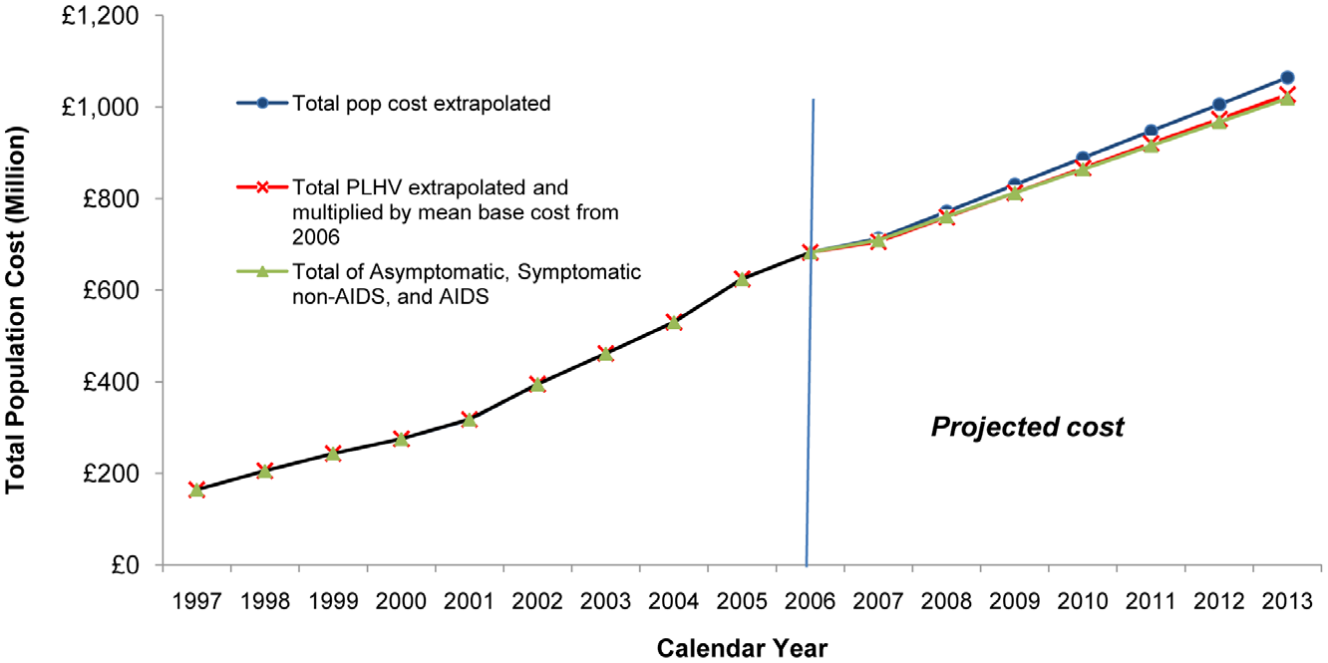
Annual Average UK Population Cost 1997–2006 and Projections 2007–2013 based on three Scenarios all including Community Care Costs (UK 2006 prices).

The population costs increased from £104 million in 1997 to £483 million in 2006 (Figure 4). If an estimate for community care costs was included, population costs rose from £164 million in 1997 to £683 million in 2006 (Figure 5).

In 1997 the total population cost for treating asymptomatic PLHIV was estimated at £22 million which increased to £162 million in 2006, while proportionally their annual cost increased from 21% in 1997 to 33% in 2006. Annual treatment costs for patients with symptomatic non-AIDS also increased, from £39 million to £146 million, but less dramatically compared with asymptomatic PLHIV; proportionally this group generated 37% of population costs in 1997, which decreased to 30% in 2006. For AIDS patients, annual population costs increased from £43 million to £176 million in 2006, but proportionally costs generated by this group decreased from 42% in 1997 to 36% by 2006.

#### Projected Annual Population Costs 2007–2013

The *first* scenario, which projected the annual population costs from 1997–2006 estimated that by 2013 the annual direct population cost would increase to £758 million or £1,065 million if community care costs were included (Figures 4 and 5).

The *second* scenario was based on the projected increase in total number of PLHIV by 2013. Multiplying the estimated number of PLHIV using NHS services by 2013 by the average 2006 per patient-year costs, the annual population cost was estimated to increase to £727 million by 2013, or £1,027 million when including community care costs (Figures 4 and 5).

For the *third* scenario, the number of PLHIV by stage of HIV infection were projected and multiplied by the average annual 2006 cost for each stage and a total annual population cost of £721 million was estimated by 2013, or £1,019 million including community care (Figures 4 and 5).

## Discussion

This study estimated that the direct population cost for treatment and care of PLHIV in the UK has risen 4.6 fold between 1997 and 2006, from £104 million to £483 million respectively. The number of PLHIV using NHS services during this period tripled from 16,075 to 52,083 PLHIV: the greatest increase was seen among asymptomatic PLHIV and less so among PLHIV with symptomatic non-AIDS or AIDS.

There has been an increase in mean annual IP days and DW visits among AIDS patients, while number of OP visits declined. Increased use of IP services was also observed among PLHIV with symptomatic non-AIDS while asymptomatic PLHIV used more DW services over time. These increases in annual cost of treatment and care over time were most pronounced for AIDS patients on quadruple-or-more ART. This may be due to these ART-experienced patients having to use more IP and DW services, as well as their increased use of new and more expensive ARVs in the second half of the study period.

The majority of patients were seen in London clinics and this may have skewed our findings, though overall costs did not seem to differ substantially between London and out-of-London sites during the study period [9]. This analysis is furthermore contingent on the fact that most if not all PLHIV using NHS services during the study, were diagnosed and reported to the HPA. Examples were described in the methods section of how some of the HPA data had to be adjusted because of missing data.

When the annual population costs were projected using three different methods, the estimated population costs increased to between £721 million and £758 million by 2013, a 1.5 fold increase from the 2006 baseline. The three scenarios produced similar estimates, with only a 5% difference between the lowest and highest estimates. The accuracy of these estimated projected figures of PLHIV using NHS services were compared with more recently figures published by HPA [12]. The recent HPA figures were 56,377, 57,930 and 62,018 for 2007, 2008 and 2009 respectively; the estimated figures were 53,842, 57,930 and 62,018 for respective years, an underestimate of 5% for each year.

Another potential source of error for estimating the population cost was the extent that HIV- services also used generic, non-HIV services to operate. While the unit costs include facility-level overheads, other general costs covered by the NHS, such as its drug procurement system, general staff training and other more general non-HIV indirect support, may not have been included in these estimates. However, these points suggest that the estimates produced in this study are likely to underestimate the true population cost of delivering HIV services in the NHS.

At the end of 2008 the number of people living with HIV in the UK was estimated to be 83,000, of whom an estimated 27% were unaware of being infected [3]; 7,298 people were newly diagnosed with HIV in 2008, or 9% of the estimated 83,000 PLHIV in the UK and 12% of the 61,213 PLHIV reportedly using NHS services during 2008. A recent study in the UK, confirmed the benefit for starting ART early with CD4 count <350 cells/mm^3^ [13]. The annual cost of treatment and care, for those who started ART with a CD4 count >200 cells/mm^3^, is 30–35% less than for those who start ART with a CD4 count ≤ 200 cells/mm^3^. However, between 1996 and 2006, of 5,541 PLHIV who started first-line therapy, 55% were diagnosed with a CD4 count ≤ 200 cells/mm^3^, many of whom were Black Africans [13]. It is likely that many of the PLHIV, who are currently unaware of being infected, may well present late in their disease course with a CD4 count ≤ 200 mm^3^. Starting more PLHIVs with a CD4 count <350 cells/mm^3^ will increase the number of people receiving HAART, which will initially add to the financial burden of the NHS. However, starting PLHIV on cost-effective regimens earlier, will maintain them in better health, resulting in the use of fewer health or social services and thereby generating fewer treatment and care costs, while enabling them to remain socially and economically active members of society.

Increasing population costs for HIV treatment and care are raising serious concerns in high-income countries, especially as many are going through periods of cutting public expenditure [14] as a consequence of the global economic downturn. This issue is even more pertinent for middle- and lower-income countries. As part of universal access, many of these countries have been increasing treatment and care services and the number of PLHIV currently on ART has reached 5 million PLHIV [15]. While this constitutes a great success, if countries want to continue to increase their ART coverage the issue of providing sustainable quality services in the context of limited country resources raises serious questions. Lessons from high-income countries teach us that increased coverage is going to result in increased population costs. Will these countries be able to provide ART free-at-point of delivery on a life-long basis, as is the underlying assumption in high-income countries [16]? The recent change in WHO criteria to start ART when CD4 count <350 cells/mm^3^ [17] increases the number of PLHIV in need of starting on ART, and also raises the ethical issue whether PLHIV with more severe HIV disease should receive ART first, or should PLHIV with higher CD4 counts have preference because their treatment and care costs are less than those with higher CD4 counts? Starting ART early is effective [18] and cost-effective [8], [19] in resource limited settings and if ART is focused on these PLHIV, one could argue that there will be more resources available that will enable more PLHIV to be receiving ART, while allowing them to remain socially and economically active. These are some of the issues, which countries and resource-limited ones in particular, will have to face. Furthermore, many low-income countries will need to make these choices with the knowledge that many of them, at least for the foreseeable future, will continue to be reliant on donor agencies or countries to sustain their treatment and care services [20].

Trying to curtail the costs of service provision is one measure by which one could try and curtail the population cost. Measures which are being used range from sending laboratory test results by email, home delivery of the drugs to the patient, which is exempted from VAT in the UK, to using the most cost-effective regimens [13]. Even if the costs could be brought down, without reducing the quality of services provided, the fact that the number of new people being infected with HIV continue to outpace those being put on ART, will continue to drive up population cost for HIV services.

Only greater prevention efforts will reduce the number of people becoming infected with HIV. A recent study from the US suggests that even if incidence is reduced drastically, the number of people newly infected with HIV will continue to increase [21]. While putting PLHIV on ART will reduce their infectivity and contribute to reducing the incidence of people newly infected with HIV [22], this in itself will not be sufficient to reduce incidence in a large number of settings. Only comprehensive prevention strategies, responding directly to the epidemic dynamics operating in each country, will be able to reduce HIV incidence [23]. It is now recognized that in most instances a combination of prevention interventions are going to be required to achieve significant reductions in HIV-incidence [24]. While recent biomedical interventions - like male-circumcision [25], the development of a vaccine [26], microbicides [27] and pre-exposure chemoprophylaxis [28] – constitute important advances, they are going to be most successful when juxtaposed with relevant behavioral and structural changes [24]. One of the findings of the Second Independent Evaluation of the UN Joint Programme on HIV/AIDS highlighted the relative lack of success of global prevention programmes, including in high-income countries, and recommended a greater emphasis on making prevention more effective and efficient [29]. Policy makers and other relevant stakeholders need to use evidence-informed HIV prevention, treatment and care strategies to ensure that PLHIV have full access to the treatments that they need based on their clinical condition, which will prolong life, reduce morbidity, reduce transmission and ultimately deliver the best for both the individual and public health agendas.
